# A coarse-graining account of individuality: how the emergence of individuals represents a summary of lower-level evolutionary processes

**DOI:** 10.1007/s10539-023-09917-x

**Published:** 2023-08-14

**Authors:** Pierrick Bourrat

**Affiliations:** 1 Department of Philosophy, Macquarie University, North Ryde, NSW, 2109 Australia; 2 Department of Philosophy and Charles Perkins Centre, The University of Sydney, Sydney, NSW 2006 Australia

**Keywords:** Individuality, Coarse-graining, Evolutionary transitions in individuality, Emergence

## Abstract

Explaining the emergence of individuality in the process of evolution remains a challenge; it faces the difficulty of characterizing adequately what ‘emergence’ amounts to. Here, I present a pragmatic account of individuality in which I take up this challenge. Following this account, individuals that emerge from an evolutionary transition in individuality are coarse-grained entities: entities that are summaries of lower-level evolutionary processes. Although this account may *prima facie* appear to ultimately rely on epistemic considerations, I show that it can be used to vindicate the emergence of individuals in a quasi-ontological sense. To this end, I discuss a recent account of evolutionary transitions in individuality proposed by Godfrey-Smith and Kerr (Brit J Philos Sci 64(1):205–222, 2013) where a transition occurs through several stages, each with an accompanying model. I focus on the final stage where higher-level entities are ascribed a separate fitness parameter, while they were not in the previous stages. In light of my account, I provide some justification for why such a change in parameters is necessary and cannot be dismissed as merely epistemic.

## Introduction

Individuals lie at the heart of evolutionary biology. They are the bearers of traits and fitness, the latter of which measures the evolutionary success of those traits. Because it is fundamental to evolutionary theorizing and practice, one might assume that individuality is defined rigorously in biology or, at the very least, that theorizing the individual is not captured entirely by the phrase ‘I know it when I see it.’ However, most biologists rely on such a vernacular concept of individuality. In many cases, this attitude is harmless and does not pose any problems. However, in other cases, particularly when individuality plays a significant role in the biological explanation one seeks to make, this reliance on the vernacular concept appears baseless or unprincipled, rendering such explanations problematic.

In the last 20 years or so, the philosophical literature has taken notice of this problem, and the work on biological individuality has blossomed.^1^ Some progress has been made in capturing the depth of this concept; however, much remains to be done. For example, it is fair to say that there is no consensus in the literature regarding how individuality should be conceived. One reason for this is that the notion of individuality is invoked in a number of biological subdisciplines that do not refer to the same underlying concept. This has led to a form of pluralism regarding biological individuality (Sterner 2015; Godfrey-Smith 2013; Chen 2015; Love and Brigandt 2017; Lidgard and Nyhart 2017b; Bueno et al. 2018a; DiFrisco 2019). While I will not contend that the term ‘biological individuality’ can mean different things in different contexts, recognizing this does not exempt us from attempting to relate and potentially unify some of these different uses and concepts wherever possible.

In this paper, my project is to provide an account of individuality within an evolutionary context. From an evolutionary perspective, individuals are evolved entities. For example, multicellular organisms did not exist until several events of so-called evolutionary transitions in individuality (ETIs) occurred at the origin of life (Buss 1987; Maynard Smith and Szathmary 1995; Clarke 2014; Griesemer 2000; Okasha 2006). One of the last, although not the last, of these was the evolution of multicellularity from unicellularity (see Maynard Smith and Szathmary 1995; Bourke 2011). The fact that individuals are evolved objects is not trivial and should be fully integrated into a mature concept of biological individuality. Even primordial individuals must have been the result of evolutionary processes that might have looked different from these modern counterparts (Bourrat 2014; Blute 2007; Wilkins et al. 2012).

Starting from this constraint, and after reviewing some of the limitations of existing proposals, I will present a new account of individuality. According to this account, individuals are coarse-grained entities (hereafter, ‘collectives’) that summarize evolutionary processes occurring to lower-level entities (hereafter, ‘particles’). Further, whether a collective is regarded as an individual depends on two things. The first is whether seeing it as such leads to a reduction in computational and/or measurement costs associated with a particular prediction. The second is whether such a reduction permits predictions that would not have possible without it.

This account brings several benefits to the table. One is that it provides a non-reified account of levels of individuality and a clear articulation between them. Each partitioning of particles into collectives can potentially be regarded as a level, but we choose some partitionings over others due to their computational or measurement efficiency and predictive power. Another related benefit is that it allows one to ground the idea of individuality empirically. At first glance, this account might appear to be merely epistemic or, more precisely, pragmatic or contextual—collectives are considered individuals because, in some contexts, it is useful to do so. While my account is resolutely pragmatic, it permits discriminating explanatory contexts where ascribing a ‘quasi ontology’ to higher-level entities as if they are independent of the lower-level entities constituting them is warranted from those where it is not.

To build this account, I start from the influential model of an ETI in terms of a shift from multilevel selection 1 (MLS1) to multilevel selection 2 (MLS2). I argue that this account faces problems if the MLS1/MLS2 distinction is interpreted as a factual one. It also faces another problem if the distinction is interpreted purely pragmatically as stemming from different modeling choices, as proposed by Godfrey-Smith and Kerr (2013). Then, I briefly present the idea of coarse-graining in general terms and show that this concept has been deployed usefully to make sense of the idea of multiple realizability. From there, I show how coarse-graining can similarly be used to account for individuality. I then argue that pragmatic constraints from lower-level descriptions represent a reason why higher-level individuals are classically granted a quasi-ontological status. Finally, I apply my account to ETIs—I revisit the work of Godfrey-Smith and Kerr (2013) and provide an interpretation of the shift between MLS1 and MLS2.

## The difficulty of accounting for the emergence of individuality

According to numerous authors (e.g., Maynard Smith and Szathmary 1995; Buss 1987; Michod 1999; Bourke 2011; Godfrey-Smith 2009; Okasha 2006; Griesemer 2000), modern organisms like us are the outcomes of evolutionary processes and the result of a succession of ETIs. Roughly, following Bourke (2011), these transitions are (in reverse chronological order) from unicelled to multicelled organisms, from asexual unicelled to sexual unicelled organisms, from prokaryotic cells to eukaryotic cells, and, finally, from separate replicators to replicators enclosed in a genome.^2^ Following an influential model (hereafter, the ‘fitness decoupling’ model), the emergence of a new level of individuality during an ETI represents a transition from an MLS1 to an MLS2 process (see Okasha 2006; Folse and Roughgarden 2010; Michod 2005).

The MLS1/MLS2 distinction was proposed by Damuth and Heisler (1988) to account for two distinct ways to characterize multilevel selection. The distinction boils down to the way collective fitness and other traits are measured and the evolution of which type of entities (particles or collectives) is tracked.

Following an MLS1 scenario, the fitness of a collective is measured in terms of number of particles produced, and characters are attributed to particles only. Thus, collective fitness (hereafter, MLS1–collective fitness) is an aggregative property of the fitness of the particles constituting a collective. In other words, collective fitness is necessarily tied to particle fitness via a mapping relationship. For example, in a collective comprising four particles, each producing two particles, the fitness of the collective under an MLS1 scenario, if measured as the average particle fitness, would be two particles. Here, the mapping between particle and MLS1–collective fitness would be the identity function. This contrasts with an MLS2 scenario where collective fitness (hereafter, MLS2–collective fitness) is measured in terms of number of collectives produced by the collective. Consequently, in such scenarios, the evolutionary fate of collectives is tracked independently from that of the particles constituting them (Damuth and Heisler 1988; Okasha 2006). While, in some MLS2 cases, particle and collective fitness (and, consequently, their respective evolutionary fates) will go hand in hand because a mapping relationship between particle and MLS2–collective fitness exists, this relationship is only a contingent one. This implies that in other cases, they will come apart. For example, a collective that produces eight particles might only produce two collectives, while another collective that produces six particles might produce three collectives. In such cases, a difference in particle fitness might not translate into a difference in collective fitness or exhibit an opposite relationship. Consequently, increasing particle fitness (or MLS1–collective fitness) might lead to a decrease in MLS2–collective fitness. This type of phenomenon is at the basis of the fitness-decoupling model for ETIs.

Okasha (2006, 104–107) provides some justification for why a change in fitness at the particle level does not necessarily translate into a change in the same direction for MLS2–collective fitness and, consequently, why particle-level and collective-level selection can be regarded as objectively independent from one another in some MLS2 cases. One main constraint for Okasha’s account is that it remains consistent with (reductionist) physicalism to prevent the emergence of a new level of selection (or individuality) from appearing mysterious. To satisfy this constraint, and yet be consistent with a contingent relationship between particle and MLS2–collective fitness, Okasha argues that collective fitness, following physicalism, necessarily depends on some particle *properties*. However, these properties are not necessarily *particle fitnesses*. Thus, one can maintain that in MLS2 scenarios relevant for the completion of an ETI, MLS2–collective fitness and particle fitness (or MLS1–collective fitness) are independent as a matter of objective facts without violating physicalism.

The observation that collective fitness becomes ‘decoupled’ or contingent from the fitness of the particles that constitute them has received some experimental support (see Hammerschmidt et al. 2014); thus, it seems to fit Okasha’s factual distinction between MLS1 and MLS2 and his model of an ETI. However, Okasha’s interpretation has been shown to be problematic as it relies on fitness measures at the two levels made in different environments (Bourrat 2015a, b; Bourrat et al. 2022; Bourrat 2021, in press). Once fitness measures at the two levels are made in the same environment, at equilibrium, fitnesses at the particle and collective levels are necessarily commensurable with one another. If fitness at the particle and collective levels are always commensurable, this poses a problem for a factual interpretation of the ‘fitness decoupling’ model of ETIs and the MLS1/MLS2 distinction since they both rely on the independence between particle and collective fitness.

In contrast to Okasha’s view, Godfrey-Smith and Kerr (2013) present a more pragmatic account of ETIs that sidesteps Okasha’s difficulty: MLS1 and MLS2 are regarded as two modeling approaches relevant for different parts of an ETI. Like Okasha’s account, MLS1 is the approach of choice in the earlier parts of an ETI, and MLS2 in the latter parts. However, contrary to Okasha, Godfrey-Smith and Kerr make no explicit commitment to whether MLS1/MLS2 should be regarded as factual or objective processes in some cases. One problem with this account is that the choice between MLS1 and MLS2 appears insufficiently justified if collectives are subsequently regarded as a genuine level of individuality. To be fair, Godfrey-Smith and Kerr point to some reasons why one might choose to model the system in an MLS1 or MLS2 way, such as that one modeling approach might give more information about the system at a particular point in the transition. Nonetheless, the last step of their account of ETIs relies on defining fitness at the collective level without reference to particle fitness. Without reference to particle fitness or an explanation of why such a reference is unnecessary, the origin of this new collective fitness, and thus individuals at that level, appears mysterious.

In the following sections, I propose an account of ETIs that draws on Okasha’s motivation to explain away the emergence of collective fitness without referring to particle fitness. One point of agreement between Okasha’s account and mine is the reliance on physicalism, which implies that a change at the lower (particle) level necessarily leads to a change at the higher (collective) level. However, my account does not suffer from the problems associated with measuring fitness in different environments, as the fitness-decoupling model does. Second, in the spirit of Godfrey-Smith and Kerr’s account, my account is grounded in pragmatic considerations. However, contrary to their account, it provides a bridge between particle-level and collective-level properties in a way that is compatible with physicalism and articulates the idea of modeling choices. Finally, my proposal provides an account of why once an ETI has occurred, it is warranted in most contexts to consider higher-level entities *as if* they were individuals, to which I refer as ‘quasi-ontological individuality.’

An important concept upon which my account relies extensively is the notion of coarse-graining. In the next section, I provide an introduction to the concept before presenting my account in subsequent sections.

## Coarse-graining

The idea of ‘coarse graining,’ as I show below, is connected to the notion of multiple realizability; thus, it is relevant in the context of multilevel systems. However, it should be clear that it applies more generally to any situation where a setting can be described more coarsely. Conceptually, coarse-graining is quite straightforward. Perhaps the simplest example thereof is to round up or down a set of numbers. Suppose that you are measuring the size of bat wings in a population in centimeters with two decimals. Then, you decide to round those numbers to the nearest integer. In doing so, you have discarded some information, effectively coarse-graining your data. Depending on the question you aim to answer, coarse-graining your data in such a way might have little to no impact on the answer you want to obtain. In other cases, when small differences in wing size make a substantial difference, rounding to the nearest integer would be problematic. For instance, in deterministic chaotic systems, such as Lorenz systems, which are highly sensitive to initial conditions, rounding can have dramatic effects over time on the evolution of the system, as Lorenz himself discovered when re-running simulations with rounded numbers (see Oestreicher 2007).

Another intuitive example of coarse-graining, which is closer to the kind of coarse-graining I deploy for my account of individuality, is to reduce the size of an image or compress it by applying a downsampling method. A very crude way to do so is to take the average pixel value of two by two squares of pixels in an image and create a new compressed image that is four times smaller. Following another algorithm, known as ‘decimation,’ one could take only the value of every fourth pixel of the initial image to create the compressed image. These two approaches would lead to different results because different bits of information would be discarded in each case. Depending on the original image resolution and the algorithm used, the human brain recognizes when too much information has been discarded, and the image appears pixelated or blurry. Modern methods of image compression aim to discard information in such a way that the human brain cannot detect it or can detect it as little as possible (for a discussion of these methods, see Gonzalez and Woods 2018, chap. 8).

These two intuitive examples demonstrate the pervasiveness and, some might argue, the triviality of coarse-graining processes. Humans routinely discard some information that is judged too fine-grained for a particular aim or to see the big picture rather than the minute details of a phenomenon. Thus, one might wonder about the significance and relevance of coarse-graining to account for scientific or philosophical concepts, such as individuality. One area where coarse-graining has played a major role is theoretical physics; an extensive formalism has been developed to study a given system from different scales. In particular, under an analysis called ‘renormalization group,’ involving coarse-graining the behavior of a system by discarding some of its details, it has been shown that very different fluids or magnets exhibiting different structures at the micro level behave similarly when described at the macro level around critical points during phase transitions (Batterman 2001). This is significant because it shows that some systems that appear very different when observed from one perspective can appear very similar when observed at a different (larger) scale. This property, called ‘universality’ in physics, has been extended beyond physics in the study of cellular automata (Wolfram 2002). Without coarse-graining a system, it would never have been possible to see the similarity in these different systems’ behaviors.

As argued by Batterman (2000), the explanatory strategy of renormalization group analysis used for universality in statistical mechanics essentially ‘eliminates degrees of freedom (microscopic details) that are inessential or irrelevant for characterizing the behavior of the system at criticality’ (p. 127)—that is, it coarse-grains the system. This strategy provides a principled way to approach the notion of ‘multiple realizability,’ which philosophers have struggled to characterize rigorously. In effect, a multiply realizable property is simply the philosophical equivalent of a universal property in statistical mechanics. In contrast, a realizer of a multiply realizable property corresponds to a description with more details. Seen through the lens of renormalization group analysis, there is nothing mysterious about the articulation of the two levels. They are merely descriptions of the same systems according to two perspectives, with the macro-level description necessarily containing less (or, at best, as much) information than the micro-level one. However, the information from the higher-level description is more relevant for the particular aim or prediction of the observer.

I argue that clarifying the idea of multiple realizability by articulating different levels of description and connecting it to the idea of universality is not the only explanatory insight to be gained from explicitly using the notion of coarse-graining. In the next sections, I deploy the idea of coarse-graining in the context of individuality. To do so, I propose a generic algorithm that can be used to decide when a set of lower-level entities should be considered an individual. In the last two sections, I tackle the problem of the emergence of higher-level individuality from a pragmatic rather than strictly ontological perspective.

## Individuals as coarse-grained entities

In this section, I start by briefly presenting two accounts of individuality that rely heavily on the idea of coarse-graining. Krakauer et al. (2020) and Libby et al. (2016) both propose a framework for defining individuality where a set of (micro)variables referring to lower-level entities is coarse-grained into a set of macro-variables referring to higher-level entities representing candidate higher-level individuals. From there, inspired by these accounts, I provide a philosophically richer and technically simpler account of individuality that is grounded in the notion of coarse-graining.

To present Krakauer et al.’s framework, suppose a set of (micro)variables in a particular state and coarse-grain these variables into two macro-variables: the ‘system’ and the ‘environment.’ Assuming a setting with *n* micro-variables, there are *a priori* $2^{n}-2$ possible ways to partition these into two macro-variables.^3^ Each of these partitionings represents one way to coarse-grain the setting of micro-variables into two macro-variables. The core of Krakauer et al.’s proposal is that when the coarse-grained system represents an individual, one should be able to predict the state of the system at a particular time horizon from the state of the system at an earlier point in time.^4^ The more accurate the prediction, the higher the degree of individuality of the system. To assess the degree of the predictability of the system between two times, Krakauer et al. invoke a number of mutual information measures. Mutual information is a quantity related to the concept of Shannon entropy (Shannon 1948; Cover and Thomas 2006). Conceptually, it is a way to measure the extent to which two variables are associated when the variables are nominal. Each of the measures proposed by Krakauer et al. represents a way to characterize individuality in a slightly different way (using different variables and conditioning on those variables), which I will not summarize here. The general idea underlying these measures is that if the mutual information of the system between two times is high following a particular coarse-graining, the system represents an individual. From there, one can provide a ranking of different coarse-grainings with different levels of individuality for the system.

Libby et al. (2016) apply another coarse-graining framework to the problem of individuality, which they call ‘state space compression’ (see Wolpert et al. 2017). Following their approach, when micro-level variables can be coarse-grained into a smaller set of macro-level variables and nonetheless yield accurate predictions, given some limits in computational resources, the higher-level entities to which those macrovariables refer can be considered individuals.^5^ To illustrate their approach, they consider a simple model where two species of fungi and two species of algae can interact and form lichens. They show that when computational resources are limited, describing the dynamics of the population of algae and fungi at a coarse-grained level can be effective for predicting the future state of the system at the expense of some accuracy in the prediction.

There is one main difference between the approaches of Krakauer et al. and Libby et al. The former only consider predictive accuracy as a constraint for characterizing individuality. In contrast, the latter add computational and measurement costs as a feature of their account. As we shall see, I argue that these costs are an important aspect to take into consideration.

With this brief presentation in place, I aim to achieve three things. First, I propose an account of individuality that retains the main insights developed by Krakauer et al. and Libby et al. without entering into the nitty-gritty details of a particular setting. To that end, my account will be presented mostly informally with the help of toy examples. Second, both Krakauer et al. and Libby et al. steer clear of any ontological implications. One of my aims is to show that an account of individuality grounded in the idea of coarse-graining can help provide some clarity about ontology and explanation. My third aim is to show the practical value of this account by deploying it in the context of previous philosophical work on ETIs.

In a population of particles, any of their properties can be coarse-grained into higher-level properties of higher-level entities. To illustrate my account beyond evolutionary settings, I will start with a non-evolutionary toy example (that could nonetheless be integrated into an evolutionary setting) that is visually appealing. Then, I will show how the account can be made relevant in an evolutionary context.

**Fig. 1:**
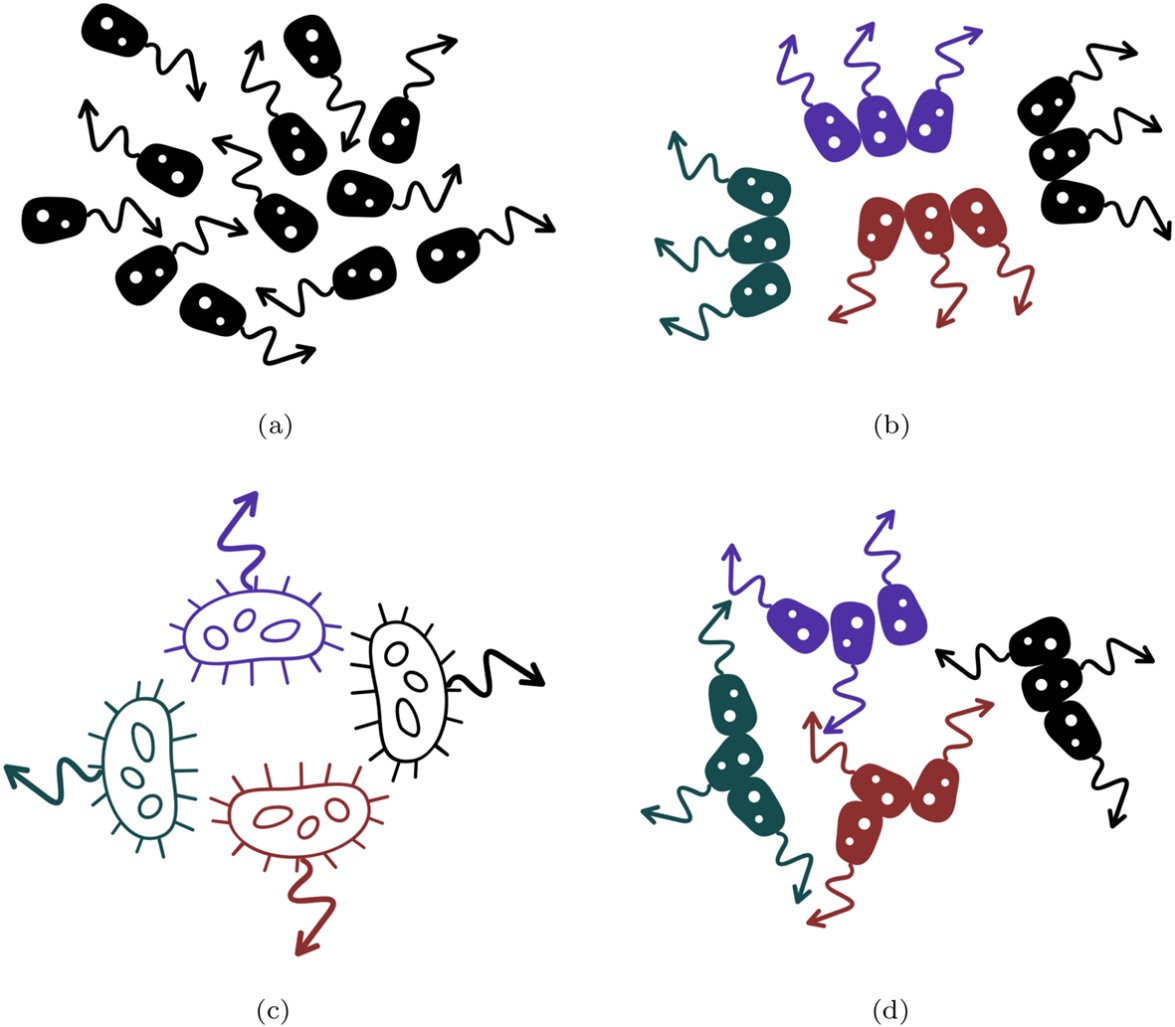
Schematic representation of a population of entities (unicellular organisms) where a coarse-graining is applied to their direction, depicted by an arrow. In **a**, no coarse-graining is applied. In **b**, entities in close proximity are coarse-grained into collectives of three particles represented by the same color. Since the entities within a collective have similar directions, it becomes pragmatically useful to consider that each collective of three is a single larger entity with its direction being the average of the directions of the particles composing them, as shown in **c**. In contrast, because the entities in close proximity have very different directions, a coarse-graining where a shift in level of individuality is assumed is not pragmatically useful in **d**. See explanations in the main text

Suppose a population of entities that could be thought of as cells, such as the one presented in Fig. 1a. We want to know whether these entities are best conceived as unicellular organisms or multicellular organisms.^6^ To answer this question, we can resort to the idea of coarse-graining and apply the following simple algorithm. We start by applying an arbitrary coarse-graining to the population of unicells (partitioning the population of unicells into larger collections of them) and assess whether, despite some information being discarded, we can still make a good prediction regarding the state of the system at a later point in time. In this particular case, coarse-graining involves taking subsets of the population and lumping the unicells within each subset by averaging their properties. If the coarse-graining leads to an accurate prediction, following this account, the coarse-grained entities can be considered individuals. If the coarse-graining performs poorly, it is not warranted to consider the coarse-grained entities individuals.^7^ We can apply this algorithm iteratively for different combinations and numbers of lower-level entities to test whether there are some individuals in a given population and whether there are different levels of individuality.

There are many cases where lower-level entities interact more substantially with distant entities, such as nervous, hormonal, or immune cells. However, a first and reasonable approximation in the context of simple or ancestral multicellular organisms is that the cells in close proximity interact with one another more often than they interact with other cells.^8^ By making this assumption, one can eliminate a number of coarse-grainings where the subsets of the population comprise entities that are far from each other and, consequently, unlikely to yield higher-level individuals.

To fix ideas, suppose we choose the direction of each particle as the property to be coarse-grained, represented by the arrows stemming from it. Under some settings, once a coarse-graining has been chosen following some rules, if we take the average direction of the entities within each subset, the coarse-grained result will appear not too different from that of each of the entities within a subset. This is the case in Fig. 1b, where a population of 12 cells has been coarse-grained into four higher-level entities, marked with different colors, as represented in Fig. 1c), each comprising three cells. As can be seen, the directions of the three red cells in the lower part of the figure starting from the left are 240$^\circ$, 220$^\circ$, and 200$^\circ$, respectively. Taking the average leads to 220$^\circ$, which is the direction ascribed to the larger entity in Fig. 1c. This means that the maximal error made by this description in absolute values for a given entity within this subset is 20$^\circ$. If we contrast this result with the same coarse-graining but in a different population, as presented in Fig. 1d, the three red entities at the same position have directions of 350$^\circ$, 240$^\circ$, and 80$^\circ$, respectively. This leads to an average value of approximately 223$^\circ$. In this case, the maximal error in absolute value for this coarse-grained description when compared to the lower-level description is 143$^\circ$.

**Fig. 2:**
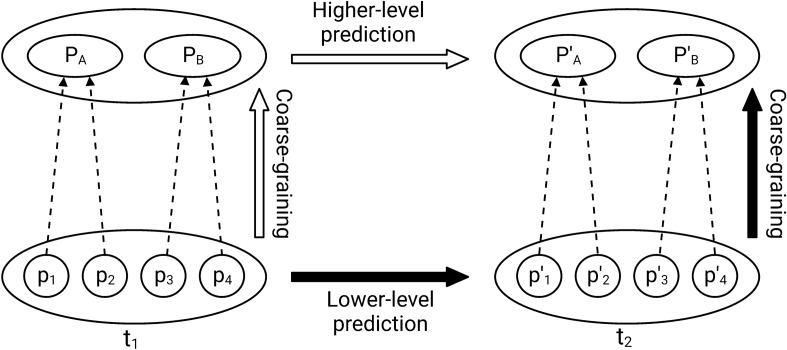
Illustration of the pathways by which a truthful and a projected prediction for a system (black and white arrows, respectively) can be made. To make a truthful prediction, one starts by evolving the system between $t_1$ and $t_2$ using properties ($p_1$, $p_2$, $p_3$, etc.) and then coarse-graining its properties at $t_2$ (from $p'_1$, $p'_2$, $p'_3$, etc. into $P'_{A}$, $P'_{B}$, etc.). In contrast, to make a projected prediction, one starts by coarse-graining the properties of the system at $t_1$ (from $p_1$, $p_2$, $p_3$, etc. into $P_{A}$, $P_{B}$, etc.), and then evolving it between $t_1$ and $t_2$ from the coarse-grained, higher-level properties. See main text for explanation

We intuitively have a sense that the coarse-graining used in Fig. 1b is more adequate than that in Fig. 1d.^9^ One way to make sense of this is to recognize that coarse-graining will be valuable in a dynamic system when it permits discarding some details but still obtains an adequate prediction of the system at a later point in time.^10^ To make an analogy with the example of coarse-graining an image presented earlier, it is valuable to coarse-grain an image if one can predict what the original image was. If not, we would not use compressing algorithms. Starting with a maximum error of 20$^\circ$ at $t_1$, as in Fig. 1b, the prediction of the displacement of the higher-level entities at $t_2$, while erroneous to some extent, will nonetheless be more accurate than if we were using the coarse-graining depicted in Fig. 1d. If the prediction errors are regarded as sufficiently small, we may even be tempted to consider that the coarse-grained entities are individuals and not refer anymore to the lower-level entities, often even forgetting that they are what fully determines the higher-level entity. How can we make this idea more precise and operationalize it?

One way to do so is to compare two predictions of the coarse-grained system’s state for a particular trait at $t_2$, one using data from the lower-level description and the other only from the higher-level description. The difference between these two predictions can then be used as an indication of whether the coarse-graining is fit for purpose or leads to unacceptable levels of error in prediction. To illustrate this, consider the situation shown in Fig. 2. We want to predict the state of the population with respect to a trait at $t_2$ from the description of the higher level. There are two possible ways to do this, represented by the two pathways with the black and white arrows in this figure, respectively. First, following the pathway with the black arrows, we start from the lower-level description at $t_1$, make a prediction of the state of the population at $t_2$ from this description, then coarse-grain the population into higher-level entities and measure the trait. This prediction is a *truthful* coarse-grained prediction of the population’s state at $t_2$. Second, following the pathway with the white arrows, we also start from the lower-level description at $t_1$ but immediately coarse-grain the population. The coarse-grained state at $t_1$ is then used to predict the coarse-grained state at $t_2$. This prediction is a *projected* prediction of the population’s coarse-grained state at $t_2$ because it is based on a prediction that left out some details.^11^

Whether a prediction of the state of the population at the higher level at $t_2$ is deemed adequate will depend on the difference between the truthful and projected predictions. If the difference is small or negligible, the coarse-graining will be harmless and the higher-level entities can be considered as if they were true entities or individuals even though they are constructions. If, however, the difference is important, considering those entities as if they were true entities will be misleading. The level of error between the truthful and projected coarse-grained prediction that will be regarded as tolerable will depend on several factors, including context and measurement errors.

Slightly more formally, we can define the difference ($\delta _C$) between the truthful prediction ($C_T$) and the projected coarse-grained prediction ($C_P$) of a given coarse-graining for the change of a trait between two times as the error in prediction:

1 $$\begin{aligned} \delta _C=C_T - C_P. \end{aligned}$$

Then, we can define a threshold $\theta$ so that:

2 $$\begin{aligned} \begin{aligned}&\text {if } \delta _C < \theta , \text { the coarse-graining is acceptable} \\&\text {if } \delta _C \ge \theta , \text { the coarse-graining is unacceptable}. \end{aligned} \end{aligned}$$

We can also define a particular $\delta _C$ in Equation (1) that is both minimal and inferior to $\theta$. This $\delta _C^*$ would allow us to characterize the best coarse-graining to predict the state of the population at $t_2$ ($C_P*$) and would be the best candidate for defining a level of individuality.

To illustrate how $\delta _C$ could be used, let us start from the examples presented in Fig. 1. In the case of the coarse-graining in Fig. 1b, assuming that the unicellular organisms within a coarse-grained entity all keep the same direction between $t_1$ and $t_2$ and all have the same velocity, we would find that $\delta _C$ for predicting the distribution in space of the coarse-grained entity from $t_1$ to $t_2$ would be small. Following the white pathway in Fig. 2, the prediction using a single coarse-grained variable for direction would tell us that the entity is at an exact position when, in fact, the entity would be distributed in space using the black pathway. This difference ($\theta$) might be acceptable for the phenomenon studied; thus, a representation in terms of collective-level individuals as in Fig. 1c might be warranted. However, if we now were to use the coarse-graining presented in Fig. 1d, following the white pathway, one would predict once again that the coarse-grained entity is at a single point when, in fact, following the black pathway in Fig. 2, it might be massively distributed in space. This discrepancy between $C_P$ and $C_T$ might be judged unacceptable, particularly if, in the phenomenon studied, spatial interactions between cells have important consequences for the dynamics of the system.

At this point, a few things should be noted. First, it should be clear that coarse-graining and finding a $\delta _C^*$ for a single trait will often be insufficient to determine whether its associated coarse-graining defines individuals unless this trait is highly correlated with many other traits. An individual is often conceived as an entity that is functionally integrated (Huxley 1912; Godfrey-Smith 2009; Santelices 1999; Hull 1980; Lidgard and Nyhart 2017b), implying that the coarse-graining used to define individuals in a population should be the same for multiple traits. Following the matrix notation in linear algebra, we can define the difference between truthful and predicted prediction as $\varvec{\delta }_{\varvec{C}}$, where the bolding indicates that they refer to vectors containing *n* traits. A coarse-graining for which a $\delta _C^*$ exists only for a single or a handful of traits could hardly be considered as defining individuals. In contrast, if a $\varvec{\delta }_{\varvec{C}^{\varvec{*}}}$ exists and refers to the same coarse-graining, one could be more confident that this coarse-graining defines higher-level individuals.

To give an example of why using a single trait or handful of traits might be insufficient to define higher levels of individuality, we could easily imagine a setting where different sub-populations of unicellular entities behave *as if* they were part of a single entity for some traits or in some particular conditions but do not for other traits or in other conditions. In the case of the toy example presented above, we could imagine that the direction of the single cells is due to positive chemotaxis for some particular resource, that this resource happens to be distributed patchily in the environment at each of the four corners of the figure, and that it diffuses in the milieu. In this case, single cells would follow the gradient of resources; naturally, the single cells closer to one another would appear as behaving together as if they were part of a multicellular entity (in this particular situation for this particular trait). However, we could easily imagine that for a different trait, such as negative phototaxis with light diffusing from the centre of the figure (or positive chemotaxis for a different chemical toward resources at the corners), the single cells that appeared to behave together in the previous case would not for these other traits. Consequently, a particular optimal coarse-graining for one trait will not be effective for another trait. In contrast, if a collective of cells does represent a multicellular organism, the optimal coarse-graining for a particular trait should remain effective, if not optimal, for other traits.

Second, in many cases, more than one $\varvec{\delta _C^*}$ could be equal to predict the state of the population at $t_2$. In such cases, the coarse-grainings they refer to could be tested for longer and shorter timesteps, and one could assess whether they perform better than the others for these different timesteps. In all likelihood, it is implausible that different coarse-grainings yield the same level of errors at all timescales.

Third, following from the previous remark, individuality might be defined over different timescales. It is expected that in a vicinity of timesteps, some coarse-grainings will fare better than others. However, when timesteps vary widely, it is implausible that the same coarse-graining will continue to be an adequate representation of the system. Therefore, new ways to coarse-grain the population (and, thus, define individuals) might be used instead, often leaving out more information about the system.

Fourth, some coarse-grainings could produce very low $\varvec{\delta _C^*}$ despite the fact that the coarse-grained entities defined by them would generally not be regarded as individuals because low $\delta _C$ are driven purely by ecological factors rather than by direct interactions between the lower-level entities. The example of chemotaxis where the single cells appear to be behaving in a concerted way due to the particular distribution pattern of the resources (presented above) is a good example of this phenomenon. In such cases, some measures of $\delta _C$ conditioned on the environment, $\varvec{\delta _C|E}$, where $\varvec{E}$ is a vector representing different aspects of the environment, could be devised. From there, one could assess whether $\varvec{\delta _C|E}$ remains consistently below the threshold $\theta$ in all the states of the environment (with the probability to be in a particular state chosen from the actual probability of the system). Several ways exist to operationalize the idea of $\varvec{\delta _C|E}$ as a distance to be compared to $\theta$. For instance, one operationalization could be the distance in a multidimensional space with each element of the vector being on one dimension; another would be to choose the element of $\varvec{\delta _C|E}$ with the highest value.

Having illustrated my account with a non-evolutionary example, I now show how it can be deployed in a multilevel evolutionary context. To begin with, note that an evolutionary process is paradigmatically a birth and death process. Thus, when following the process between two times, new particles might be created, and some existing ones might die. Similarly, some collectives might be created and others die. Because the birth (or death) of a particle does not necessarily lead to the birth (or death) of a collective, additional rules for mapping the two would be necessary. Figure 2 only depicts objects that transform their properties between $t_1$ and $t_2$. To illustrate an evolutionary process, one would also have to depict the production and death of objects.

Following this remark, coarse-graining a population of particles into collectives and tracking the evolution of this population from the perspective of collectives requires two steps. First, one needs some mapping rules to define collectives from particles, as in the non-evolutionary case. Second, when particles are produced or die, one must have some rules that map these events to the coarse-grained description in terms of collectives.

Importantly, different rules can lead to very different outcomes in terms of collective offspring. For simplicity, we could suppose that generations at the particle and collective levels are discrete and synchronous so that any offspring particle produced would mean that it belongs to a collective offspring. Of course, this is not the case in most situations. However, I will make this assumption here as it permits me to simplify considerably the illustration.

Naturally, as with the non-evolutionary case, some coarse-grainings will fare better for predicting the dynamics of the population at a later time. To see this, suppose that to reproduce, particles must always interact with at least three other particles. A coarse-graining that would capture this would allow making an accurate prediction about collective-level reproduction and, thus, the dynamics of the system. In this case, $\delta _C$ would be small. In contrast, a coarse-graining rule that would lead to considering that collectives are always composed of two particles would see some of the collective reproducing, and some not reproducing, with no means of determining the reason for this stochastic behavior. However, this difference would be due to the fact that the coarse-graining would not have captured that among the collectives defined by this inefficient coarse-graining, some are composed of particles that interact with at least three other particles, while others are not. In that case, $\delta _C$ would be higher and might be judged too large.

Having presented an approach for defining higher individuality by coarse-graining a population of entities, how does this relate to the problem of the emergence of higher-level individuality presented in Sect. 2? It should first be noted that the coarse-graining account makes it transparent that its conception of a level is primarily a level of *description*. Following this view, the higher levels are merely summarized descriptions of the lower levels. There is nothing that exists at the higher level that could not be described or predicted, in principle, for the lower level. Whether such predictions are possible, in practice, is an important question to which I will soon turn. However, this bears no consequence for the conceptual point I make. What remains to be explained is how the proposal made in this section permits one to account for the quasi-ontological status of higher-level individuals. All my claims thus far have remained in the epistemic realm. I have shown that one *can* use a higher-level description of lower-level phenomena, rather than why one *should* or, put even more strongly, why it is indispensable to do so in some contexts. I turn to this question in the following two sections.

## Pragmatic considerations and the quasi-ontological status of higher-level individuals

Thus far, my treatment of individuality in terms of coarse-graining has not considered any pragmatic aspects. Without doing so, one might question the advantages of coarse-graining a population of entities into larger ones, knowing full well that any prediction from coarse-grained entities will typically lead to prediction errors.^12^ Why not always use truthful predictions from the lower level? Doing so would never lead to any prediction errors.

On first pass, one might respond that a fine-grained description is not always the optimal way to describe a system for the question asked. However, one counter-response is that by describing the system finely, one always has the choice to discard information and coarse-grain lower-level description if this is required by the type of question asked. The reverse process, fine-graining from a higher-level description in situations where this would be required, is not possible without making new measurements or assumptions. This is so because coarse-grained descriptions, assuming they refer to the same substrate, always contain less information than more fine-grained ones.^13^ Thus, the asymmetry between fine-grained and coarse-grained descriptions anchors the primacy of lower-level descriptions over higher-level ones.

A more promising reason is that coarse-graining reduces the dimensionality of a system. If measurement and computational power are limited (as they always are in our finite world), reducing the dimensionality of a system implies that, for the same number of operations, one can predict the state of the population for a higher number of timesteps. For instance, in an agent-based simulation (for an introduction, see Railsback and Grimm 2011), with a limit of 100 operations, assuming a population of 10 entities, each with a single property and no interaction between the entities, one can predict the state of the population, assuming a single operation per entity per timestep for 10 timesteps. By coarse-graining this population into two collective entities, each with a single property, the gain in prediction power means that with the same number of operations, one can predict the state of the system after 50 timesteps, assuming again a single operation per entity per timestep.^14^

With respect to measurement constraints, suppose an experiment such as the ones described in Hammerschmidt et al. (2014) or Rose et al. (2019), where the goal is to study the earlier stage of an ETI using a bacterial system. Making measurements at the bacterial cell level is often practically impossible within a reasonable timeframe and with resources allocated to the experiment. Instead, measurements are made at the colony level. The cost is that these measurements might not be as precise as if the measurements have been made at the bacterium level. However, the gain is that from these measurements, predictions can potentially be made. Had all the resources been allocated to make measurements of cells rather than colonies, the sample sizes for the different conditions would have most likely been too small to perform any statistical tests. Additionally, in many cases, it is not possible to measure the particles of a collective without altering the system, especially if it is highly functionally integrated. Such alterations make it difficult—in some cases impossible—to obtain reliable subsequent measurements. Measurements at the collective level become the only possible ones. Thus, measurement constraints also exist in systems where particle number is small, such as bee hives or ant colonies, when compared to the bacterial system mentioned above.

These simple examples lead to a deeper point. Although coarse-graining often comes at the cost of precision in prediction, this ‘cost’ is often a counterfactual one. In the above example about computational costs, the prediction obtained after 50 timesteps might be less accurate by coarse-graining the population than if a prediction from after 50 timesteps had been made using lower-level properties. However, making such a prediction would have required a computational power of 500 operations. With no access to such a number of operations, the coarse-grained description is the only viable one. Further, note that, in practice, the choice between a lower-level and higher-level description of a system might not exist; one might only have access to the higher-level description. In such cases, there is no micro-level description where measurement and computational costs could *actually* be paid to make predictions at these levels. Consequently, higher-level predictions are made without reference to the lower level even if one could agree that, in principle, making predictions from more fine-grained data would lead to better predictions.

This latter point is, I believe, an important reason why higher-level individuals, such as multicellular organisms, are often regarded as having an ontological status in biology. They stand as *sine qua non* entities to make predictions or provide explanations, and this property is sufficient to see them as having an ontology. If one were attempting to describe their evolution from a lower-level (cellular) perspective, all the measurement and computational resources deployed to fulfill that aim would only yield predictions over much shorter timescales. For longer-term predictions on the phylogenetic tree of life,^15^ the resolution used must change. ETIs are the placeholders for these changes in resolution, and they become indispensable when the measurement and computational costs incurred become higher than can be afforded. ETIs lie at the breaking point where a pragmatic but indispensable shift in description must occur.

## Multilevel selection 1 and 2 and pragmatic constraints

The view developed in the previous section relates to and provides an extension of the model of ETIs proposed by Godfrey-Smith and Kerr (2013).

Recall from Sect. 2 that the solution to the problem of the emergence of higher-level individuality proposed by Okasha (2006) as a transition from MLS1 to MLS2, where MLS1 and MLS2 are seen as holding a contingent rather than necessary relation, faced an important difficulty. The difficulty is that when fitness at the particle and the collective level are compared in the same reference environment, a change in the fitness of a particle necessarily leads to a change in the fitness of the collective it constitutes. Starting from previous work (see Kerr and Godfrey-Smith 2002), Godfrey-Smith and Kerr (2013) propose an account of ETIs that eschews this difficulty; their account is devoid of any ontological interpretation and is rather presented as a modeling exercise. Their account comprises five stages, each of which can be described using a model with different state variables (i.e., the variables used to track the changes in the system) and parameters, such as fitness values for particles. I focus on the fourth and fifth ones since the previous three stages are fully accountable by an MLS1 description—that is, what they call a ‘gestalt switching’ between particle-level and multilevel description is possible.^16^ Briefly, in the first stage, particles start interacting with one another, but there are no collectives. In the second stage, collective formation occurs, and particles interact within collectives. In the third stage, collectives become more cohesive.

In the fourth stage, it becomes more difficult to explain the change observed in terms of particles. During that stage, the population of particles is organized into functionally integrated collectives, of which the evolutionary dynamics becomes best accounted for by a model where state variables are changed from particle-level to collective-level variables. Such a change could be motivated, as Godfrey-Smith and Kerr argue, by the collective phase of the life cycle of the organism studied becoming more prominent than in earlier stages. This stage initiates the transition in MLS2. While, as we have seen in Sect. 2, in an MLS1 scenario, the state variables always ultimately refer to the particles, in an MLS2 scenario, one switches to tracking the frequency of collectives over time. However, note that solely tracking the frequency of collectives is not sufficient for the scenario to be of the MLS2 kind, where MLS2 represents something more than a convention. For an MLS2 setting where gestalt switching is not possible, collective fitness *must be* measured in terms of collectives produced rather than particles, with no possibility of reference to particle fitness. This represents Godfrey-Smith and Kerr’s fifth stage. Thus, schematically, in the fourth stage, instead of the model tracking the evolutionary success of the particles between timesteps, the focus becomes the collectives. However, this success is still measured in terms of offspring particles produced. In the fifth stage, evolutionary success becomes measured in terms of offspring collectives produced, with a new fitness parameter that bears no necessary relationship to the fitness of particles; thus, a shift from MLS1 to MLS2 has occurred.

While I am generally in agreement with Godfrey-Smith and Kerr’s treatment, it leaves two important points open to question. 1) Why would one start tracking collectives rather than particles? 2) Why would one need to switch the level at which fitness is ascribed? Godfrey-Smith and Kerr do not provide definitive answers to these questions and resist any reference to emergence or similar terms. Rather, they propose the choices and interests of the modeler as a guide. While choice and interests are certainly important,^17^ they fundamentally exist in the epistemic realm. Thus, their proposal does not account for why ETIs are regarded as things happening in the world rather than in the mind of the modeler. However, as Griesemer (2008, p. 1328) makes very clear: ‘Individuality concepts thus have a dual character: they are partly about processes in the world beyond and around us and partly about us, in so far as our concepts are linked to the mode and manner of our tracking engagements with the world.’ I propose that measurement and computational constraints, following the coarse-graining account provided above, permit us to articulate this dual character.

To begin with, it should be emphasized that for most of their treatment (the first three stages), Godfrey-Smith and Kerr assume that the particle-level and collective-level descriptions contain the same quantity of information. In other words, at each stage where gestalt switching is readily achievable, the quantity of information is the same at both levels. This effectively means that, in those stages, no coarse-graining from the lower to the higher level is occurring. Only when a switch from MLS1 to MLS2 is initiated, during the last two stages of their account of ETIs, do they relax the assumption of the same quantity of information between the two levels, thus leaving some scope for coarse-graining making a difference for prediction as some information is discarded.

In the fourth stage, they assume that when switching to tracking collectives rather than particles, some information to recover collectives from particles might be missing. This can be explained by the fact that, over a life cycle involving collective-level and particle-level stages, one might measure the state of the system at different points in the life cycle, and some states might be derivable from other states but not vice versa. This has interesting implications about coarse-graining: the higher-level (collective) phase might have a higher dimensionality than the lower one and, consequently, that coarse-graining could also be made from a higher-level to a lower-level description. However, it should be clear that 1) ‘higher’ and ‘lower’ levels refer here to different temporal stages rather than to a mereological relationship, and 2) their account does not require this information to be missing. I will not discuss this assumption further here.

In the fifth stage, Godfrey-Smith and Kerr also relax the assumption of an equal quantity of information at both levels of description in a way that is more relevant to my purpose here. They assume that fitness is solely defined at the collective level with new parameters, which coincides with the transition being achieved and MLS2 becoming ‘primitive’ rather than derived from particle properties. The collective fitness parameters introduced do not necessarily bear any relationship to the fitness of the particles composing them. Thus, at that stage, a map between particle and collective fitness is not provided.

In addition to citing the choices of the modeler as reasons why such a map is not provided, I argue that in most real situations, the primary reason is that this information is fundamentally unknown, either because it is simply not measured or because it would be too costly to do so for a prediction at the desired time horizon. When this happens, tracking collectives and their properties rather than particles is the only pragmatic solution to adopt. Once the transition has occurred, it becomes only possible to explain the fate of the system in collective rather than particle terms. Thus, understood that way, there is no *factual* change of the level at which selection occurs during an ETI, as is proposed in Okasha’s model. However, for pragmatic reasons, it becomes useful to pretend as if this were the case—hence, the ‘quasi ontological’ label. Thus, while Godfrey-Smith and Kerr point to pragmatic considerations as *choices* made by the modeler, I emphasize that, complementarily, *constraints* due to computational and measurement costs are important aspects of switching from a lower to a higher level of description. Godfrey-Smith and Kerr could obviously respond that by ‘choice,’ they also meant constraints in my sense; in this case, my analysis in terms of coarse-graining would provide a rationale for their account.

The view proposed here also relates to one of Godfrey-Smith’s 2006 discussions of the strategy of model-based science. In it, he argues that science sometimes progresses from building models where some of the elements of the model are ‘imagined concrete things’ that bear some resemblance to the target system and correspond to the ‘folk ontology’ of many scientists. By giving individuals the status of imagined objects, one can make sense of an ETI as a change in ontology without having to explain how it occurs causally, since what can happen in the mind of a modeler or scientist does not have to be consistent with what happens physically. Once an ETI is complete, due to the measurement and computational costs that would come with a lower-level description, modelers switch ontology and consider higher-level entities as primitive rather than derived from the lower level and start building models either formally or more verbally from this assumption.

To conclude this section, the interpretation of ETIs in terms of a shift from MSL1 to MLS2 as resulting from pragmatic constraints rather than being a factual shift in levels of selection provides a simple interpretation of the gap between particle-level and collective-level fitness. Following the coarse-graining account proposed here, it is unsurprising that measures of fitness at the particle and collective level correlate with one another at the beginning of a transition but not necessarily once the transition is complete: at the beginning of the transition, the two levels contain the same quantity of information (and gestalt switching is possible). Toward the end of the transition, because the interactions between cells become computationally too onerous to track or simply because they cannot be measured, the time horizon over which they can be neglected is short and does not match the dynamics observed when a prediction using fitness at the collective level is given.

## Conclusion

In this paper, I deployed general ideas about coarse-graining in the context of individuality and ETIs. By adopting a conception of levels of individuality as being fundamentally levels of description, I argued that one can gain traction on the emergence of individuality in evolution by showing that despite individuals being ultimately epistemic tools, their ontological feel and look can be grounded in pragmatic constraints. More particularly, I argued that considering individuals as having a quasi-ontological status when lower-level descriptions are not possible is not only harmless but also allows for predictions that a lower-level description would not permit. The main benefits of this approach are that it casts some light on the transition from an MLS1 to an MLS2 process during ETIs, a view that has gained popularity in the literature, and also supposes the existence of a map between the terms at different levels of description, which permits accounting for the emergence of individuality in a non-mysterious and principled way.

## Footnotes

1 For instances of this, see the following edited collections: Calcott and Sterelny (2011), Bouchard and Huneman (2013), Guay and Pradeu (2015), Lidgard and Nyhart (2017a), Bueno et al. (2018a).

2 In total, Bourke distinguishes six types of ETIs, two of which (the transitions from multicellular organisms to eusocial society, and from separate species to interspecific mutualism) are not relevant for explaining the emergence of multicellular organisms per se.

3 This assumes each micro-variable contains at least one macro-variable. In half the combinations, the ‘environment’ and the ‘system’ would be reversed.

4 What represents an appropriate time horizon will depend on the explanatory context. Thus, if one coarse-graining provides better short-term predictions, but another provides better long-term predictions, choosing one over the other could be made based on the explanatory context.

5 Libby et al. use the term ‘organism’ rather than ‘individual.’ Since their account can potentially be applied at any level of organization and to any substrate, I believe the word ‘individual’ better characterizes the target of their account.

6 Note that we assume that the status of those unicellular entities is not questioned; they are considered *bona fide* individuals.

7 In the example, I will assume coarse-graining for a single property to illustrate the point; however, it should be clear at the outset that more than one property is necessary to assess the performance of a particular coarse-graining. I explain why below.

8 More generally, this idea could be implemented in a model by supposing cells are nodes in a network, and interactions are the edges. Coarse-grained entities that maximize the number of edges within them but minimize those between them would represent individuals.

9 To be clear, whether a coarse-graining is adequate cannot typically depend on a single trait (as we shall see) because some coarse-grainings could lead to spurious results.

10 There are clear links here—that would be worth investigating further—with the ideas of projectibility of predicates by Goodman (1954) and that of real patterns proposed by Dennett (1991). The former is discussed in relation to individuality in DiFrisco (2019). I thank Ellen Clarke for pointing out these connections to me.

11 To be clear, the distinction between truthful and projected prediction is only relative to the assumptions I made for this particular model. I assumed that the lower-level entities objectively represent individuals; however, they could also themselves be the result of coarse-graining at lower levels. Note also that one potential problem for making a projected coarse-grained prediction is that a well-defined functional mapping between the lower-level and higher-level description does not exist. I assume here that it exists, or that, because the higher level mereologically supervenes on the lower level, a higher-level description would necessarily represent a coarse-graining of *some* properties at the lower level even if the relationship between the two levels cannot be represented adequately. In such cases, the vertical white arrow of Fig. 2 would be assumed rather than represent a functional mapping between the two levels.

12 In some cases, such as when there is no variation within the larger entities, there might be no prediction errors, but these cases should be regarded as limit cases.

13 Under the assumption of (reductionist) physicalism, in some very specific cases coarse-grained descriptions might contain as much information, but never more than fine-grained descriptions if both descriptions refer to the same substrate.

14 Note that this reasoning assumes that no operation on groups of entities in the population, such as ‘ask all red entities to do X,’ have been made. This is so because such a grouping already represents a form of coarse-graining.

15 Which certainly involves many events of reticulation.

16 This idea of gestalt switching comes from the literature on pluralism or conventionalism about levels of selection (for a short review, see Bourrat 2021), according to which there is no fact of the matter as to which level natural selection operates at in an MLS1 setting.

17 For reasons why they are, see Bueno et al. (2018a) and, in particular, the contributions of Bueno et al. (2018b), Waters (2018), Love (2018), and Griesemer (2018).
